# Impact of Mt. Olympus Honeys on Virulence Factors Implicated in Pathogenesis Exerted by *Pseudomonas aeruginosa*

**DOI:** 10.3390/antibiotics12060998

**Published:** 2023-06-01

**Authors:** Eleni Tsavea, Paraskevi Tzika, Eleni Katsivelou, Anna Adamopoulou, Marios Nikolaidis, Grigorios D. Amoutzias, Dimitris Mossialos

**Affiliations:** 1 Laboratory of Microbial Biotechnology, Molecular Bacteriology-Virology, Department of Biochemistry & Biotechnology, School of Health Sciences, University of Thessaly, 41500 Larissa, Greece; elenats89@hotmail.com (E.T.); tzikap@food.ihu.gr (P.T.); elenikatsivelou@gmail.com (E.K.); ann.oulita@hotmail.com (A.A.); 2 Bioinformatics Laboratory, Department of Biochemistry & Biotechnology, School of Health Sciences, University of Thessaly, 41500 Larissa, Greece; mnikolaidis@uth.gr (M.N.); amoutzias@uth.gr (G.D.A.)

**Keywords:** Mt. Olympus, honey, honey bee products, virulence factors, elastase, pyoverdine, biofilm, motility, Pseudomonas aeruginosa

## Abstract

The aim of this study was to examine the impact of twenty honey samples, harvested in Mt. Olympus (Greece), on the virulence factors implicated in *P. aeruginosa* pathogenesis. Six key virulence factors (protease and elastase activity, pyocyanin and pyoverdine concentration, biofilm formation, and swimming motility) were selected in order to assess the effect of the tested honeys compared with Manuka honey. All tested honeys demonstrated a significant inhibition of protease and elastase activity compared with the control. Six and thirteen honeys exerted superior protease (no inhibition zone) and elastase (values lower than 55%) activity, respectively, compared with Manuka honey. Seventeen tested honeys exhibited reduced pyoverdine production compared with the control; all tested honeys, except for one, showed an inhibitory effect on pyocyanin production compared with the control. Regarding swimming motility, nine tested honeys demonstrated significantly higher inhibition compared with Manuka honey. Honey concentrations (6% *v/v* and 8% *v*/*v*) had the most profound impact, as they reduced biofilm formation to less than 20% compared with the control. Overall, our data demonstrate a significant inhibition of the virulence factors in the tested Mt. Olympus honeys, highlighting the strong antimicrobial activity against *P. aeruginosa,* an antibiotic-resistant pathogen of growing concern, which is implicated in severe nosocomial infections globally.

## 1. Introduction

*Pseudomonas aeruginosa* is a gram-negative, aerobic, rod-shaped bacterium of the *Pseudomonadaceae* family that is present in diverse environments including soil, plants, and animal tissue [1]. It is an opportunistic pathogen, causing acute and chronic infections, wound infections, as well as lung infections related to cystic fibrosis [2,3]. Furthermore, multidrug-resistant (MDR) *P. aeruginosa* strains are of growing concern, implicated in numerous severe nosocomial infections globally [4].

The mechanisms underlying *P. aeruginosa* infections are complex, involving the production of extracellular and intracellular virulence factors that contribute to pathogenicity [5]. These factors are secreted by *P. aeruginosa* and include enzymes such as alkaline protease and elastase, toxins such as exotoxin A, pigments such as pyoverdine and pyocyanin, structural components such as lipopolysaccharides, as well as components related to motility, such as pili and flagella [6]. Pyocyanin production leads to increased oxidative stress and modifies the host mitochondrial electron transport system [7], while pyoverdine, the main siderophore produced by *P. aeruginosa,* has been demonstrated to be a virulence factor in different host models [8]. Furthermore, *P. aeruginosa* forms a structure consisting of cells embedded in an exopolysaccharide matrix, designated a biofilm, thus further contributing to resistance to the host’s immune response and antibiotics [9].

The polar flagella are responsible for swimming motility, while surface structures such as Type IV pili perform important functions such as adherence to different surfaces (eukaryotic cells and abiotic surfaces) and mediate a different mode of movement called twitching motility [10]. *P. aeruginosa* elastase, encoded by *LasB*, is one of the major proteins and is the dominant protease secreted by many strains of this opportunistic pathogen [11].This enzyme is involved in tissue damage and degrades a variety of proteins in the blood, including immunoglobulins, coagulation and complement factors, and α-proteinase inhibitors [12].

During the last decades, there has been a growing interest in implementing alternatives to antibiotics in order to combat drug-resistant pathogens. Honey has long been used to treat wounds and infectious diseases [13]. It is a sweet mixture of various substances, including glucose, fructose, and other sugars as well as proteins, enzymes, vitamins, minerals, and phytochemicals such as polyphenols [14,15]. Recent studies have documented well the in vitro and in vivo antibacterial and antibiofilm activity of different types of honey against pathogenic bacteria as well as the effect of honey on quorum sensing and bacterial motility [16,17,18,19,20,21,22,23,24,25]. Previous studies have reported that the antibacterial activity of honey is attributed to a combination of factors, such as hydrogen peroxide (H_2_O_2_), low pH, methylglyoxal (MGO), antimicrobial peptides, and osmotic stress [26,27,28].

Due to the increased resistance of *P. aeruginosa*, novel drugs or natural products need to be implemented in order to enhance the effectiveness of current therapies. In that context, we previously examined the antibacterial activity of honeys produced in Mt. Olympus against *P. aeruginosa* [29]. Mount Olympus in Greece is renowned for its rich plant diversity due to its unique climate as a high mountain (2918 m) near the Aegean Sea. The location serves as a meeting point of the Mediterranean and Central European floras, where more than 1500 plant species have been reported, including 29 endemic species [22,30]. The aim of this study was to investigate for the first time the impact of Mt. Olympus honeys on the major virulence factors of *P. aeruginosa*, which contribute to the virulence exerted by this opportunistic pathogen.

## 2. Results and Discussion

### 2.1. Impact of Honeys on Protease Activity in Pseudomonas aeruginosa

**Figure 1:**
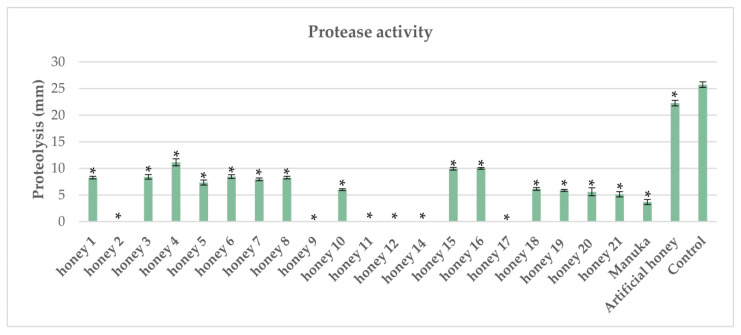
Impact of honeys on protease activity of *P. aeruginosa.* Each honey was compared against the control, and statistically significant differences are shown with an asterisk (*).

As a control for our study, we used *P. aeruginosa* proteolysis in the absence of honeys. All tested honeys demonstrated statistically significant, strong inhibition of protease activity compared with the control (Figure 1). Laboratory-synthesized (artificial) honey inhibited the protease activity of *P. aeruginosa* (control) much less than did the tested honeys (though still statistically significant), indicating that compounds other than sugars might further contribute to protease activity inhibition. Fourteen honey samples exerted lower inhibitory activity, while six honeys demonstrated superior inhibitory activity compared with Manuka honey (Figure 1). Honey exhibits antibacterial activity through distinct mechanisms, including hydrogen peroxide production, methylglyoxal, antimicrobial peptides, and osmotic stress. On the other hand, polyphenols and flavonoids have been recognized as major antibacterial compounds in honey [31]. A recent study showed that extracts of *Salix tetrasperma* (Indian willow) stem bark and flowers exhibited an inhibitory effect on the proteolytic activities of *P. aeruginosa* through the disruption of quorum sensing [32]. Another study by Yin et al. [33] demonstrated that tea polyphenols inhibited *P. aeruginosa* protease activity in a similar way. These findings document that polyphenols from plant extracts contribute to antibacterial activity against *P. aeruginosa.* Furthermore, Kafantaris et al. [22] demonstrated that pine honey strongly downregulated the expression of the *P. aeruginosa* PA14 *LasA* gene, which encodes a staphylolytic exoprotease. Therefore, it is plausible that protease activity inhibition could be attributed to the phenolic acids and flavonoids present in different types of honey through the disruption of quorum sensing, which regulates the production of several virulence factors, including proteases.

### 2.2. Impact of Honeys on Elastase Activity in P. aeruginosa

**Figure 2:**
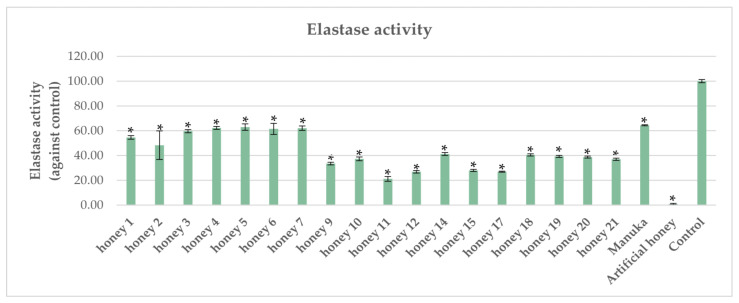
Inhibition of *P. aeruginosa* elastase activity by honeys. Each honey was compared against the control, and statistically significant differences are shown with an asterisk (*).

All tested honeys demonstrated statistically significant inhibition of elastase activity compared with the control, which was defined as 100% elastase activity (Figure 2). Five tested honey samples exhibited a similar inhibitory activity on elastase compared to Manuka, while thirteen honeys showed superior inhibitory activity. Elastase is a specialized zinc metalloprotease that is regulated by quorum sensing [34]. Recently it was demonstrated that elastase significantly contributes to the establishment of chronic lung colonization and modulates the immune response in a mouse model [35]. It has been reported that Chinese herbal extracts rich in polyphenols exerted a strong inhibitory effect on *P. aeruginosa* elastase activity [36]. Recently, a study conducted on heather and Manuka honey, using molecular docking, demonstrated that honey constituents such as benzoic acid (a product of polyphenol breakdown) and methylglyoxal (MGO) could negatively affect the activity of oxidoreductase enzyme DsbA1 [37]. The DsbA1 enzyme catalyzes the formation of disulfide bonds in a plethora of *P. aeruginosa* virulence proteins, including elastase [38]. In accordance with these findings, a recent study by Ivanov et al. [39] demonstrated that polyphenols, especially rutin, reduced the production of *P. aeruginosa* elastase. In addition to the polyphenols present in honey that could affect elastase activity by impairing disulfide bond formation, the high sugar content found in several honeys could disrupt quorum sensing, thus indirectly affecting elastase activity, as reported by Wang et al. [40]. Therefore, it is reasonable to assume that the very strong inhibition of elastase activity by artificial honey, a rather surprising finding in this study, could be attributed to its high sugar content.

### 2.3. Impact of Honeys on Pyoverdine Production in P. aeruginosa

**Figure 3:**
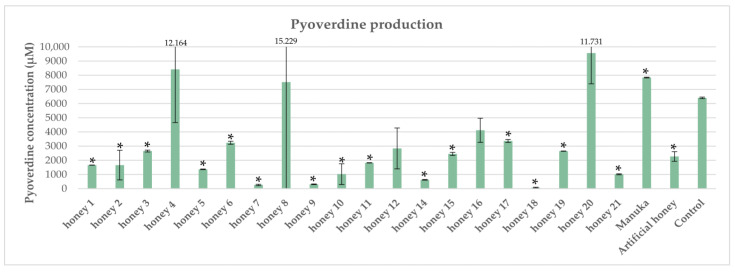
Impact of honeys on pyoverdine production in *P. aeruginosa.* Each honey was compared against the control, and statistically significant differences are shown with an asterisk (*).

Seventeen out of twenty Mt. Olympus honeys (statistically significant in the case of 15 honeys) demonstrated reduced pyoverdine production compared with the control, e.g., *P. aeruginosa* grown under iron limitation in the absence of honey (Figure 3). However, in the present study, Manuka honey enhanced the pyoverdine production, which is not in accordance with previously reported data [35]. Nevertheless, our data support the more recent findings of a study conducted by Ankley et al. [41] in which siderophore production in *P. aeruginosa* treated with diverse subinhibitory concentrations of Manuka honey was significantly greater than in the untreated control. In a previous study conducted by Kafantaris et al. [22], RNA-seq. analysis revealed that a group of genes implicated in iron uptake and transport were upregulated when *P. aeruginosa* PA14 was treated with a subinhibitory concentration of pine honey. Furthermore, two genes *(pchD* and *pchE*) that are implicated in pyochelin biosynthesis were upregulated, but not the genes implicated in the biosynthesis of pyoverdine, which is the major siderophore produced by *P. aeruginosa*. In this study, we report a significant reduction in pyoverdine production in the presence of most Mt. Olympus honeys. The observed variability regarding the level of reduction could be attributed to different concentrations and/or different qualitative compositions of the polyphenols. Polyphenols could indirectly reduce the production of pyoverdine by impairing disulfide bond formation in the key enzymes implicated in pyoverdine biosynthesis [8], as has been suggested for elastase activity. Three of the tested honeys demonstrated an enhanced pyoverdine production compared with the control. However, experimental variation was an issue in these particular honeys (as depicted by the high standard deviation values). Overall, our data suggest that different types of honey could impose an iron-limited environment for *P. aeruginosa*, which might be exploited by the application of siderophore–antibiotic conjugates as an alternative approach to combat this notorious opportunistic pathogen.

### 2.4. Impact of Honeys on Pyocyanin Production in P. aeruginosa

**Figure 4:**
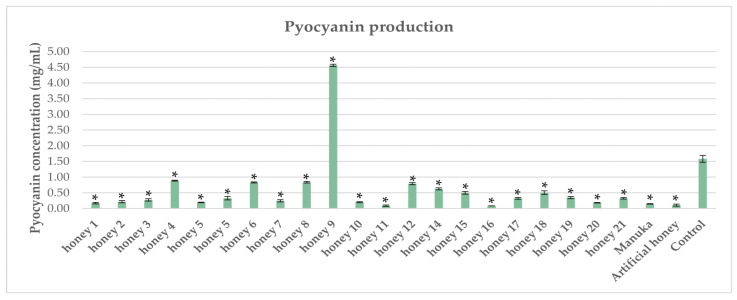
Impact of honeys on pyocyanin production in *P. aeruginosa.* Each honey was compared against the control, and statistically significant differences are shown with an asterisk (*).

All tested honeys showed a statistically significant inhibitory effect on pyocyanin production compared with the control, except for one honey (No 9). Two Mt. Olympus honeys demonstrated inhibitory activity comparable to or slightly higher than Manuka. Eighteen Mt. Olympus honeys exhibited lower inhibitory activity in comparison with Manuka honey (Figure 4). Pyocyanin is a major virulence factor produced by *P. aeruginosa* that acts as a pro-oxidant by elevating free radical levels through Fenton reaction in combination with siderophores, particularly pyochelin [8]. Our results are in agreement with a recent study conducted by Bazaid et al. [42], demonstrating that Sumra honey inhibited pyocyanin production in *P. aeruginosa* at a subinhibitory concentration through quorum-sensing disruption. It is plausible that the observed notable reduction in pyocyanin could be attributed not only to the sugar content but to the phenolic compounds and their derivatives, as has been demonstrated previously [43].

### 2.5. Impact of Honeys on Swimming Motility of P. aeruginosa

**Figure 5:**
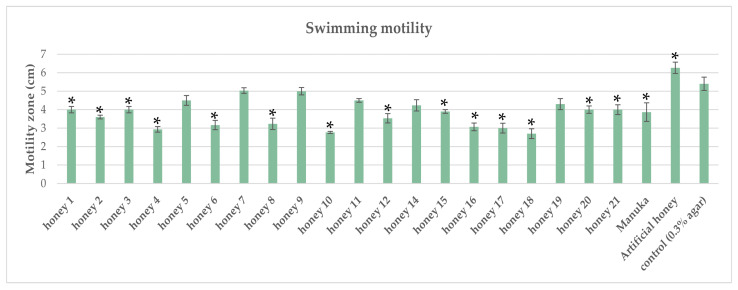
Effect of honeys on swimming motility of *P. aeruginosa.* Each honey was compared against the control, and statistically significant differences are shown with an asterisk (*).

All the tested honeys inhibited the swimming motility of *P. aeruginosa*. Nine Mt. Olympus honeys showed a higher inhibitory activity on swimming motility compared with Manuka, while eleven tested honeys exhibited a lower inhibitory activity (Figure 5). Swimming motility is mediated by flagella. Our results demonstrated that honeys could affect the function of flagella and of Type IV pili, thus resulting in the prevention of bacterial adhesion and colonization. Research conducted by Roberts et al. [2] revealed that swimming motility was negatively affected by Manuka honey treatment. Genes associated with flagella formation were downregulated in a concentration-dependent manner. This de-flagellation led to reduced motility, adherence, and eventually virulence of *P. aeruginosa* [44]. A recent study by Ye et al. [6] showed that daphnetin, a coumarin derivative extracted from plants, could inhibit the swimming motility of *P. aeruginosa*. Therefore, phytochemical honey compounds could act in a similar way.

### 2.6. Impact of Honeys on Biofilm Formation of P. aeruginosa

**Figure 6:**
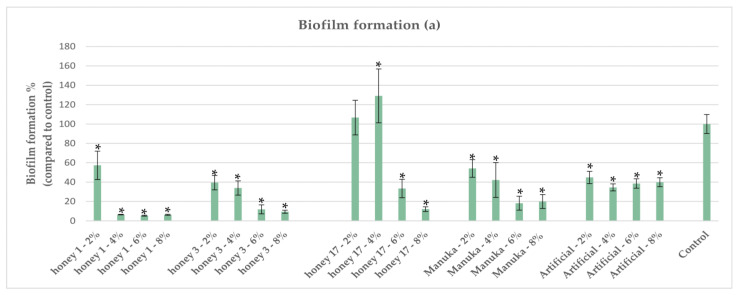
Effect of honey samples 1, 3, and 17 on biofilm formation of *P. aeruginosa.* Each honey was compared against the control, and statistically significant differences are shown with an asterisk (*).

**Figure 7:**
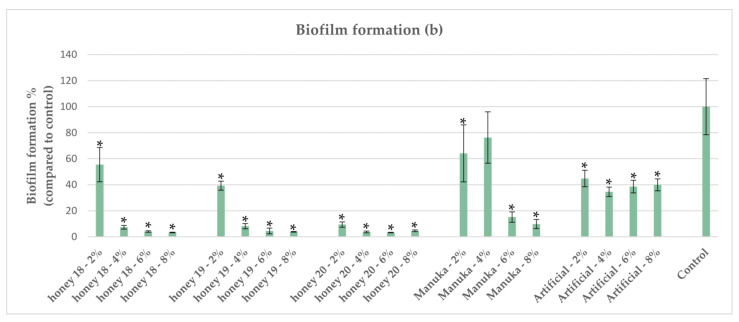
Effect of honey samples 18, 19, and 20 on biofilm formation of *P. aeruginosa.* Each honey was compared against the control, and statistically significant differences are shown with an asterisk (*).

A subset of the Mt. Olympus honey samples demonstrating comparable or superior antibacterial activity against *P. aeruginosa* compared with Manuka [29] were tested for antibiofilm activity. Honey concentrations at 6% *v/v* and 8% *v/v* demonstrated the most profound effect, reducing biofilm formation to less than 20% compared with the control in a dose-dependent manner (Figure 6 and Figure 7). All tested honeys demonstrated superior inhibitory activity on biofilm compared with Manuka at 8% *v/v* concentration. In *P. aeruginosa*, the quorum-sensing system plays a crucial role in biofilm formation, leading to increased virulence and resistance to antibiotics [44]. A study conducted on Manuka honey using a molecular docking approach demonstrated that MGO strongly binds to the *P. aeruginosa* enzyme DsbA1. This finding may explain how Manuka honey hinders the formation of *P. aeruginosa* biofilm by affecting the fimbriae and flagella [37]. Furthermore, a study by Majtan et al. [45] demonstrated that honeydew honey supplemented with vitamin C improved the antibiofilm activity against *P. aeruginosa*. Vitamin C hinders the production of extracellular polymers, which are essential for bacterial biofilm formation [46]. Recently, Pleeging et al. [25] examined the effect of six different wound care products containing medical-grade honey (MGH) on biofilm formation and eradication in *P. aeruginosa* using an in vitro wound biofilm model containing an artificial dermis. The MGH products showed significant antibiofilm activity (formation and eradication) on the artificial dermis, especially those supplemented with vitamin C and E, clearly indicating a synergistic effect. Another recent study by Farkas et al. [47] examined the antibiofilm activity of four unifloral honeys harvested in Hungary. The antibiofilm activity was variable depending on the botanical origin (in accordance with our findings), with linden honey exerting the highest biofilm degradation [47]. A study conducted in our lab [22] demonstrated that pine honey strongly downregulated genes such as *pa1L* (*lecA*) and *lecB*, which are involved in biofilm formation pathways. Proaño et al. [48] investigated the antibiofilm activity of eucalyptus honey against *P. aeruginosa* and the effect of honey constituents and indicated that the ability of this honey to inhibit biofilm formation is due to the presence of H_2_O_2_ and Def-1. They also reported that the osmotic effect of eucalyptus honey affects bacteria in the early stages of biofilm formation, causing dehydration of cells within pre-existing biofilms, leading to their death and weakening the biofilm.

*P. aeruginosa* is a highly adaptable, gram-negative, opportunistic pathogen among the ESKAPE group of bacteria [49]. It is frequently linked with hospital-acquired infections and is known to be invasive and toxin producing. The expression of numerous virulence factors involves multiple interconnected pathways that work together to regulate bacterial virulence [50]. Research has demonstrated that phytochemicals hinder the production of virulence factors that are regulated by quorum sensing, such as biofilm formation, protease, elastase, and pyocyanin [49]. A study investigating the combined effects of curcumin and natural honey on the quorum-sensing-regulated virulence factors in *P. aeruginosa* demonstrated that the production of pyocyanin, pyoverdine, and elastase was significantly reduced [51]. Another study demonstrated that benzoic acid negatively affects the virulence of *P. aeruginosa* in plants by limiting the production of virulence factors such as pyocyanin and by lowering the overall activity of protease and elastase [52]. Honey exerts antibacterial activity not only due to hydrogen peroxide and MGO, which are the major antibacterial factors, but also due to its complex chemical composition [26]. Honey contains a wide range of natural compounds, including polyphenols and flavonoids [53], which collectively contribute to its health benefits. Specific polyphenols may act synergistically with other compounds to enhance the overall antimicrobial efficacy of honey [54].

## 3. Materials and Methods

### 3.1. Honey Samples

Twenty honey samples produced in the Mt. Olympus region were donated by individual beekeepers and beekeeper associations. Every sample was assigned a unique reference number, and the information about its geographical location, botanical source, and date of collection was recorded (Table 1). The samples were stored for no longer than 18 months before analysis in glass jars at room temperature.

Manuka honey, MGO 550+ (Manuka Health, Auckland, New Zealand), was used for comparison in this study, while laboratory-synthesized (artificial) honey was included as a negative control. The laboratory-synthesized honey was prepared by mixing 3 g sucrose, 15 g maltose, 80.1 g fructose, and 67 g glucose (all supplied by Sigma-Aldrich, Athens, Greece) in 34 mL deionized and sterile water. The sugar solution was heated to 56 °C in a water bath to help with dissolving [55].

### 3.2. Bacterial Strain and Growth Conditions

The antibacterial activity of the honeys was tested against carbapenem-resistant *P. aeruginosa* 1773 (kindly provided by Professor Spyros Pournaras, School of Medicine, University of Athens, Athens, Greece), identified and characterized by standard laboratory methods. The *P. aeruginosa* was routinely grown in Mueller–Hinton broth or Mueller–Hinton agar (Lab M, Heywood, UK) at 37 °C.

### 3.3. Protease Activity Assay

The assay was performed according to Wang et al. [40]. Briefly, 4% *v/v* of honey was added to PCA (plate count agar) (Lab M, UK) petri dishes containing 1.5% *w/v* skimmed milk and then inoculated with 2 μL of *P. aeruginosa* culture at OD_600_ = 2.0. The plates were incubated at 25 °C for 48 h. Manuka honey was used as a positive control and laboratory-synthesized honey as a negative control. The diameter of the proteolysis zones in mm was recorded. The assay was carried out in triplicate, and the reported proteolysis zone diameter is the mean of the measurements.

### 3.4. Elastase Activity Assay

Tubes containing 2 mL of nutrient broth supplemented with 4% *v/v* honey were inoculated with *P. aeruginosa*. The tubes were incubated at 37 °C for 18 h at 210 rpm. A culture of *P. aeruginosa* in the absence of honey was used as a control (elastase activity was defined as 100%). One milliliter of each culture was centrifuged for 3 min at 10,000× *g,* and 50 μL of the supernatant was transferred to an Eppendorf tube containing 20 mg elastin Congo red (Sigma-Aldrich, Athens, Greece) and 1 mL Na_2_HPO_4_, pH = 7. The Eppendorf tubes were incubated at 37 °C for 4 h, and then the supernatant was measured at 495 nm [56]. The assay was carried out in triplicate, and the reported elastase activity is the mean of the measurements.

### 3.5. Pyoverdine Assay

Cultures were grown in 5 mL of King’s broth nutrient substrate [57] (50 mL of King’s broth requires 1 g of bacteriological peptone (Lab M, UK), 0.5 mL of glycerol (SERVA, Germany), 0.075 g of KH_2_PO_4_ (Alfa Aesar, Kandel, Germany), and 0.075 g of MgSO_4_·7H_2_O (Alfa Aesar, Germany) supplemented with 4% *v*/*v* honey and inoculated with 5 μL of *P. aeruginosa* culture at OD_600_ = 2.0. The vials were incubated at 37 °C for 24 h at 210 rpm. Manuka honey was used as the positive control and laboratory-synthesized honey as the negative control. Appearance of a yellow-green color is an indication of pyoverdine secretion by *P. aeruginosa*. For pyoverdine extraction, 1.5 mL of culture was centrifuged for 2 min at 10,000× *g*. The supernatant absorbance was measured at 405 nm, and then the concentration of pyoverdine was calculated based on the formula: A = e × b × C [58] (A: absorbance at 405 nm, e: molecular damping coefficient = 1.4 × 10^4^ M^−1^cm^−1^, b: 1 cm, C: concentration). Each assay was carried out in triplicate, and the yield of pyoverdine concentration is reported as the mean of the measurements ± their standard deviation.

### 3.6. Pyocyanin Assay

Cultures were grown in 5 mL of tryptone soy broth (Lab M, UK) supplemented with 4% *v/v* honey and inoculated with 5 μL of *P. aeruginosa* OD_600_ = 2.0. The vials were incubated at 37 °C for 24 h at 210 rpm. The following day, samples were taken and measured for OD_600nm_ to account for differences in cell density. Appearance of a blue-green color is an indication of pyocyanin secretion by *P. aeruginosa*. For pyocyanin extraction, 1 mL of culture was centrifuged for 3 min at 10,000× *g*. Then, 750 μL from the clear supernatant was mixed with an equal volume of chloroform. The lower blue phase was removed and mixed with an equal volume of HCl 0.2 M. A new phase separation was observed and the reddish phase was extracted and measured at 520 nm [59]. The concentration of pyocyanin was calculated from the formula mg/L = A × 17.072. Each assay was carried out in triplicate, and the yield of pyocyanin concentration is reported as the mean of the measurements ± their standard deviation.

### 3.7. Swimming Motility Assay

Luria–Bertani broth petri dishes, supplemented with 0.3% *w/v* agar and 4% *v/v* honey, were inoculated with *P. aeruginosa* culture [60]. The petri dishes were incubated for 48 h at 37 °C and the mobility zone was recorded. Each assay was carried out in triplicate, and the bacterial motility is reported as the mean of the measurements ± their standard deviation.

### 3.8. Inhibition of Biofilm Formation

*P. aeruginosa* was initially grown in nutrient broth (Lab M, UK) overnight at 37 °C. Using sterile 96-well polystyrene microtiter plates (Kisker Biotech GmbH & Co. Steinfurt, Germany), honey samples in LB (190 μL) were added at final concentrations of 8%, 6%, 4%, and 2% *v*/*v*. The overnight *P. aeruginosa* culture was diluted (1:10) in LB broth supplemented with 2% glucose, and 10 μL was added to the wells. Wells containing only LB + 2% glucose were used as negative controls. The microtiter plates were incubated at 37 °C for 48 h, allowing biofilm formation. After 48 h, the liquid phase was removed and washed three times with phosphate buffer saline (PBS). The adherent cells were fixed with methanol and air-dried at 37 °C for 20 min. Formed biofilms were stained with 0.4% *w/v* crystal violet for 15 min. The crystal violet was removed, and the wells were rinsed with sterile deionized water. After air drying, 30% acetic acid was added for 15 min, and then the detached *P. aeruginosa* cells were transferred into a new microtiter plate. The absorbance was measured at 630 nm using a microplate reader (ELx808 Absorbance Microplate Reader, BioTek Instruments, Inc., Winooski, VT, USA) [61].

### 3.9. Statistical Analysis

In each of the assays (4.2–4.8), we compared the values of the three biological replicates of each honey sample against the control sample using the SciPy Python package [62]. The comparison was performed using the two-tailed *t*-test for paired samples, and the resulting p-values were corrected using the Benjamini–Hochberg multiple-testing correction (FDR) from the statsmodels Python package [63]. Statistically significant differences are depicted with an asterisk.

## 4. Conclusions

A previous study assessed the antibacterial activity of honeys harvested in Mt. Olympus against *P. aeruginosa* in comparison with Manuka. The antibacterial activity exerted by some Mt. Olympus honeys was comparable or even superior to that of Manuka. The aim of the present study was to elucidate the mechanisms underlying the demonstrated antibacterial activity, especially the impact on the major virulence factors expressed by *P. aeruginosa*. Our data in most cases demonstrated a statistically significant negative impact on virulence factors in the presence of Mt. Olympus honeys. The reduced expression of the virulence factors might be attributed to quorum-sensing disruption and/or impaired disulfide bond formation. It is reported that polyphenols and flavonoids are the key honey constituents exerting antimicrobial activity through diverse mechanisms, which is in accordance with our findings. However, the present study demonstrated that the impact on the virulence factors was highly variable among the tested honeys. A possible interpretation of these findings could be differences (quantitative and qualitative) among the Mt. Olympus honeys regarding polyphenols and flavonoids. The rich plant diversity of Mt. Olympus could significantly affect polyphenol composition, thus explaining the differences in antimicrobial activity. Future research should focus on polyphenol analysis correlated with botanical origin in order to further elucidate the impact of specific Mt. Olympus honey constituents on the mechanistic aspects (such as virulence factors) of antimicrobial activity exerted against *P. aeruginosa*.

## Figures and Tables

**Table 1:** Botanical source, geographical location, and harvest date of the honey types.

Harvest Date	Geographical Location	Botanical Source	Honey Number
July, 2013	Karia	Polyfloral	1
July, 2013	Elassona	Alfalfa and herbs	2
July, 2013	Sikea	Polyfloral	3
July, 2013	Paliampela	Oregano and herbs	4
July, 2013	Domeniko	Polyfloral	5
July, 2013	Samina	Polyfloral	6
July, 2013	Sarantaporo	Polyfloral	7
July, 2013	Krania	Mint, herbs, and acacia	8
July, 2013	Azoro	Polyfloral	9
July, 2013	Verdikoussia	Polyfloral	10
July, 2013	Kalithea	Polyfloral	11
June, 2012	Karia	Polyfloral	12
2012	Domeniko	Polyfloral and conifers	14
July, 2014	Karia	Polyfloral	15
2012	Elassona	Polyfloral	16
2014	Drimos	Herbs	17
August, 2014	Karia	Acacia, Abies, and Sideritis	18
August, 2014	Azoros	Polyfloral and conifers	19
2014	Galanovrissi	Polyfloral and conifers	20
July, 2014	Karia	Polyfloral and conifers	21

## Data Availability

Not applicable.
